# Sympatho-adrenergic activation by endurance exercise: Effect on metanephrines spillover and its role in predicting athlete’s performance

**DOI:** 10.18632/oncotarget.24584

**Published:** 2018-02-26

**Authors:** Elisa Danese, Cantor Tarperi, Gian Luca Salvagno, Alessandra Guzzo, Fabian Sanchis-Gomar, Luca Festa, Luciano Bertinato, Martina Montagnana, Federico Schena, Giuseppe Lippi

**Affiliations:** ^1^ Laboratory of Clinical Biochemistry, Department of Neurological, Biomedical and Movement Sciences, University of Verona, Verona, Italy; ^2^ School of Sport and Exercise Sciences, Department of Neurological, Biomedical and Movement Sciences, University of Verona, Verona, Italy; ^3^ Leon H. Charney Division of Cardiology, New York University School of Medicine, New York, New York, USA; ^4^ Department of Physiology, Faculty of Medicine, University of Valencia and INCLIVA Biomedical Research Institute, Valencia, Spain

**Keywords:** sympathoadrenal system, plasma metanephrines, endurance exercise, running performance

## Abstract

**Background:**

The sympatho-adrenergic activation during exercise is implicated in many cardiovascular respiratory and metabolic adaptations which have been thought to partially explain the different levels of performance observed between trained and untrained subjects. To date, no evidence exists about the association between competition performance and markers of “acute stress response”. We designed this study to investigate; (i) the acute sympatho-adrenergic activation during endurance exercise in recreational runners by measuring plasma levels of free metanephrine (MN) and normethanephrine (NMN) before and after a half-marathon run; (ii) the association between the metanephrines levels and the running time.

**Methods:**

26 amateur runners (15 males, 11 females) aged 30 to 63 years were enrolled. The quantification of MN and NMN was performed by LC-MS/MS. Anthropometric ergonomic and routine laboratory data were recorded. Statistical analyses included paired *T*-test, univariate and multivariate regressions.

**Results:**

The post-run values of MN and NMN displayed a nearly 3.5 and 7 fold increase respectively compared to the baseline values (*p <* 0.0001 for both). NMN pre-run values and pre/post run delta values showed a significant direct and inverse association (*p =* 0.021 and *p =* 0.033, respectively) with running performance. No correlations were found for MN values.

**Conclusion:**

NMN is a reliable marker of sympatho-adrenergic activation by exercise and can predict endurance performance in the individual athlete. Adaptation phenomenon occurring not only in the adrenal medulla might represent the biological mechanism underlying this association. Further studies on sympatho-adrenergic activation, competition performance and training status should contemplate the measurement of these metabolites instead of their unstable precursors.

## INTRODUCTION

Catecholamines, namely epinephrine (adrenaline) and norepinephrine (noradrenaline), are known to play an important role in the adaptive processes in response to physical, environmental and psychological stressors throughout the activation of the sympatho-adrenomedullary (SAM) system. A considerable increase of catecholamine plasma levels has been reported in many conditions, including physical exercise [1], insulin induced hypoglycemia [2], hypoxia and hipercapnia [3], and caffeine intake [4, 5].

In normal conditions, the synthesis of norepinephrine occurs mostly within the sympathetic nervous fibre extremities and, to a lesser extent, within chromaffin cells of adrenal medulla. Unlike norepinephrine, epinephrine is almost entirely synthetized and released from the adrenal medulla gland in response to direct stimulation of sympathetic nervous system, adrenocorticotropic hormone and/or cortisol [6]. Both hormones act as messengers of the SAM system, as nonselective agonists of α (subtypes α_1_ and α_2_) and β (subtypes β_1_ and β_2_) adrenergic receptors. Norepinephrine preferentially binds to α-receptors, while epinephrine preferentially binds to β-receptors, although their selectivity depends on the circulating concentration [7, 8]. Briefly, the activation of α_1_-receptors increases total peripheric resistance (TPR), blood pressure (BP), which may elicit a reflex bradycardia, and provokes constriction of bronchial smooth muscle and gastrointestinal smooth muscles relaxation, whilst α_2_-receptors activation decrease sympathetic outflow. On the other hand, β_1_-receptor stimulation is associated with enhanced heart rate (positive chronotropy), stroke volume, cardiac output, pulse pressure, and cardiomyocyte contractility, while β_2_-receptor stimulation induces skeletal muscle arterial vasodilatation, bronchial smooth muscle relaxation, increases skeletal muscle and liver glycogenolysis, and increases lipolysis [9, 10]. Since epinephrine is metabolized to metanephrine (MN) and norepinephrine is metabolized to normetanephrine (NMN), these free circulating metanephrines (i.e., catecholamine metabolites) are commonly used to identify increases in sympathoadrenal function [11, 12].

An increased adrenergic tone leads to higher circulating catecholamines levels, which is a characteristic of the adaptation of the organism to exercise [13]. The sympathoadrenal response to physical exercise increases respiratory, cardiac, metabolic and thermoregulatory functions depending on specific exercise characteristics (i.e., type, duration and intensity). For instance, endurance exercise leads to remodeling of the heart muscle by adrenergic inputs, including an adaptive (or “physiological”) hypertrophy process [14]. Sympathetic nervous system stimulation triggers metabolic processes that increase both perfusion pressure of the coronary artery and coronary vasodilation. This is a “healthy” compensatory mechanism, necessary to maintain an increased demand [15, 16]. In effect, endurance exercise-induced changes in human heart anatomy have been extensively described (athlete’s heart) [17]. Likewise, other factors such as age, gender, nutritional and emotional status may have an influence on catecholamine release in exercising subjects [1].

Controversy remains on the influence of training status on catecholamine responses to exercise at the same absolute and/or relative intensity. Although higher post-exercise epinephrine plasma concentrations have been occasionally described in endurance and sprint-trained athletes compared to untrained subjects, or in anaerobic-trained athletes compared to aerobic-trained subjects, others studies failed to observe a significant effect of endurance training [1, 18]. Evidence collected in animal studies seems more consistent to suggest that physical training may be effective to increase epinephrine secretory capacity by inducing adrenal gland hypertrophy [19, 20].

This phenomenon, known as “sport adrenal medulla”, may partially explain the higher physical performance observed in trained compared with untrained subjects. According to this hypothesis, athletes with higher basal nocturnal catecholamine excretion (BNCE) may obtain better competitive results or athletic performance than those with lower levels. This finding has been consistently observed in competitive cross-country skiers [21], basketball players [22], sky-flyers [23], and military ongoing flight missions [24], thus suggesting the existence of a relationship between competition performance and “average sympathetic activity” of individual athletes. Nevertheless, studies evaluating the association between competition performance and “acute stress response” are still lacking to the best of our knowledge.

Therefore, we designed this study to investigate: (i) the acute sympathoadrenal activity during endurance exercise in recreational runners by measuring the plasma levels of free metanephrines, i.e., MN and NMN, before and after a half-marathon run; (ii) the association between the circulating levels of metanephrines and the running time as a reliable index of competitive athlete’s performance.

## RESULTS

**Figure 1:**
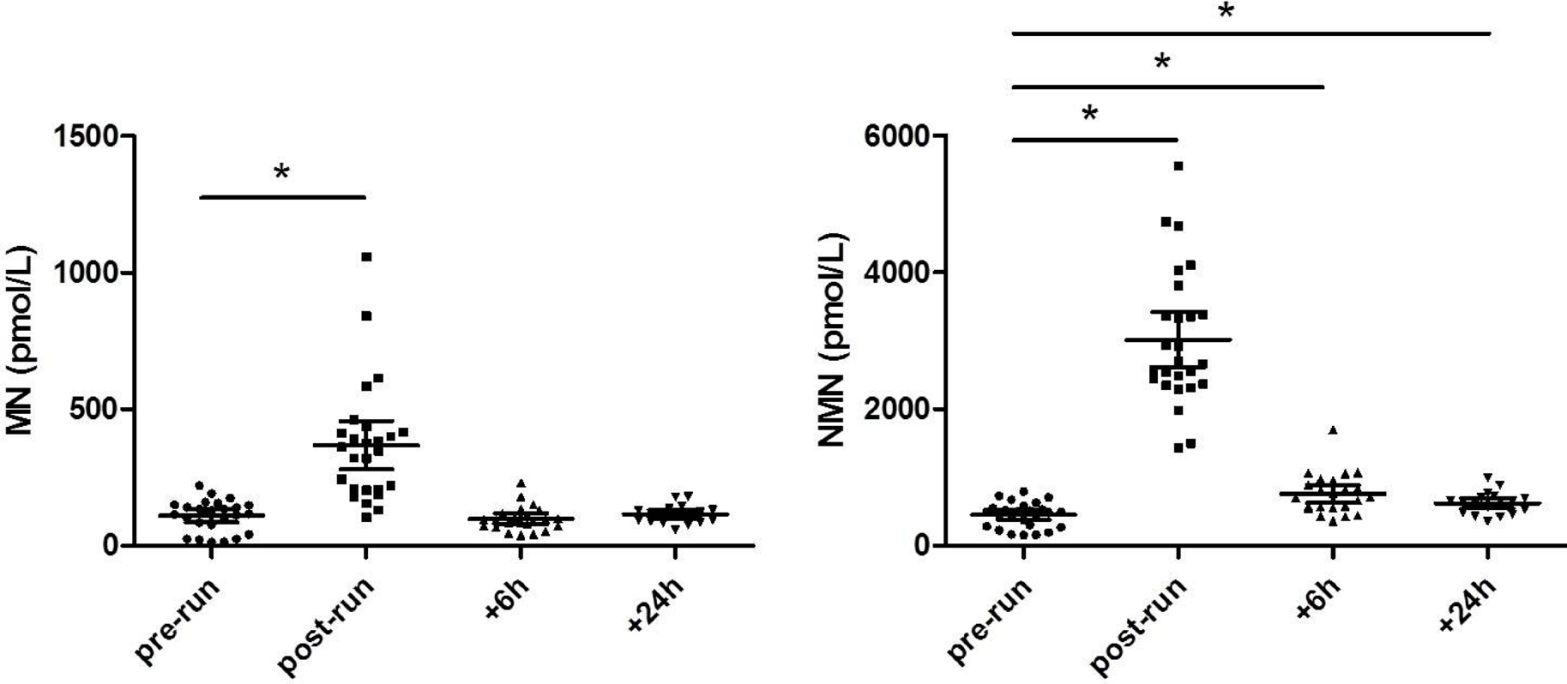
MN and NMN values at different time point before and after the half-marathon run ^*^Significant difference with respect to pre-run values.

The baseline characteristics of the athletes are summarized in Table 1. The post-run values of MN (354.5 pmol/L; range, 105–1058 pmol/L) displayed a nearly 3.5 fold increase compared to the baseline values measured before the run (118.5 pmol/L; range, 14–222 pmol/L; *p <* 0.0001) (Figure 1). Six and 24 hours after the run, the MN returned to values comparable to the basal concentration (95 pmol/L; range, 39–232 pmol/L; *p =* 0.6373 and 116.5 pmol/L; range, 59–180 pmol/L; *p =* 0.2575). The post-run concentration of NMN displayed a nearly 7-fold increase compared to the baseline values measured before the run (2685.5 pmol/L; range, 1432–5563 pmol/L vs 488.5 pmol/L; range, 158–798 pmol/L; *p <* 0.0001). Unlike MN, the plasma concentration of NMN measured at 6 and 24 h after the run remained significantly higher than the pre-run concentration (715 pmol/L; range, 362–1704 pmol/L; *p <* 0.0001 and 652.5 pmol/L, range, 359–993 pmol/L; *p =* 0.0031). Moreover, the concentration of NMN measured 24 hours after the run was lower than that measured 6 hours after the run (*p =* 0.0026).

**Table 1:** Demographical, anthropometric and ergonomic data

	Median (range)
Age (years)	47 (30–63)
Gender (M/F)	15/11
BMI (Kg/m^2^)	22.8 (18.5–27.8)
VO_2max_ (mL/kg/min)	49.5 (40.6–58.0)
Running performance (min)	112 (91–149)

VO_2max_, maximal oxygen uptake

**Figure 2:**
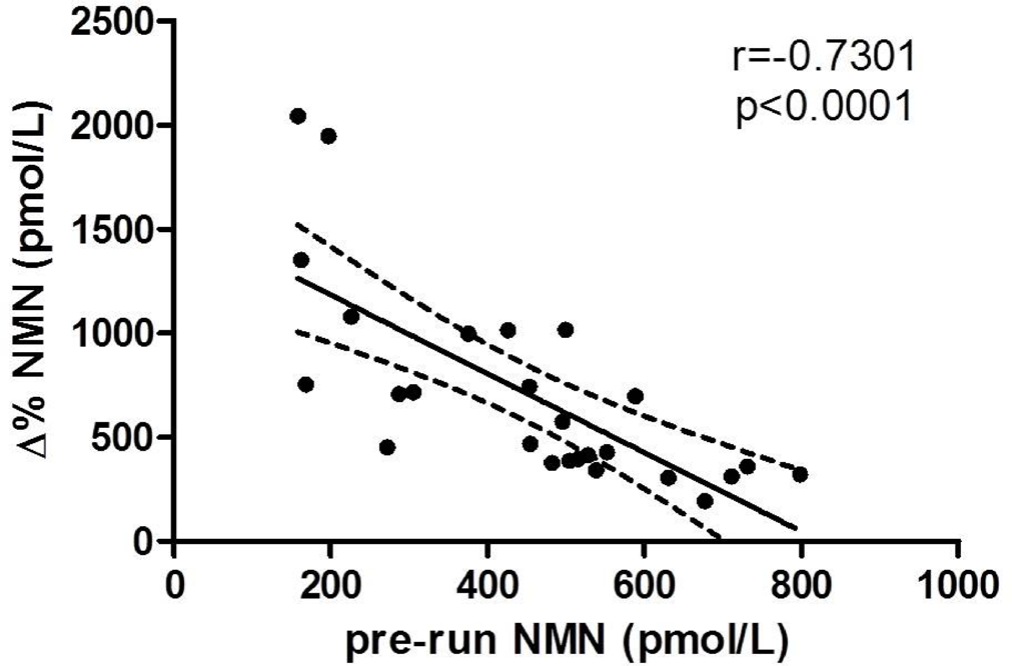
Correlation between pre-run and delta percentage values of NMN

The delta values of both MN and NMN did not significantly correlate with any anthropometric baseline characteristic or laboratory data. Nevertheless, a statically significant correlation was found between NMN variation and VO_2max_ (*r =* 0.663, *p =* 0.005). Interestingly, the pre-run values of NMN were found to be inversely correlated with NMN delta values (Figure 2).

**Figure 3:**
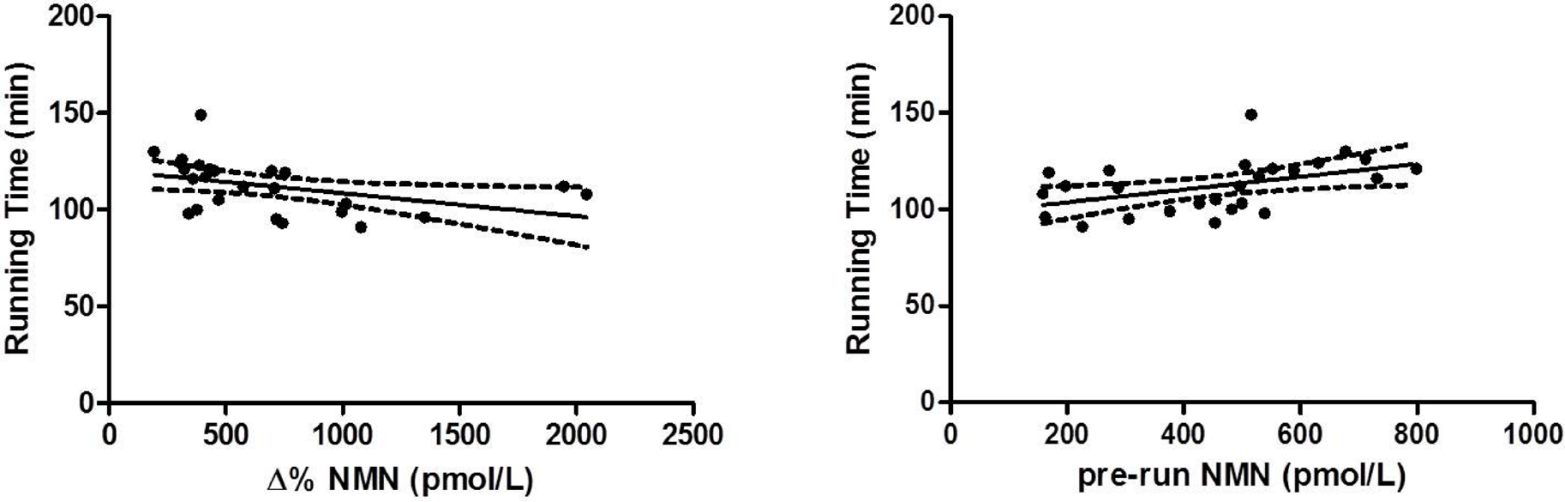
Linear regression analyses of NMN pre-run and delta values and running performance

In univariate analysis, running performance was found to be significantly associated with sex (*r =* 0.418, *p =* 0.025), age (0.300, *p =* 0.049), capillary blood lactate (*r =* –0.590, *p =* 0.001), pre-run NMN values (*r =* 0.451, *p =* 0.021) and NMN delta values (*r =* –0.418, *p =* 0.033). These two last associations are shown in Figure 3. In multivariate analysis, where running time was entered as a dependent variable and the parameters significantly associated with running performance in univariate analysis were entered as independent variables, all parameters were confirmed to be significant predictors of running performance (Table 2). Due to the correlation between delta and pre-run NMN values, the former was not entered in the multivariable model. The combination of gender, sex, age, blood lactate and delta values of NMN predicted 82.2% (95% CI, 73.8–90.6%; *p <* 0.001) of variance in running performance.

**Table 2:** Multivariate analysis showing independent predictors of running performance

Independent variables	β coefficient	*P* value
Age (years)	0.75	0.003
NMN ∆% (pmol/L)	–0.012	0.004
Gender	–11.4	0.008
Capillary blood lactate (mmol/L)	–2.6	0.023

## DISCUSSION

The biology of sympatho-adrenergic activation in response to exercise has been a matter of debate for almost 30 years. Available evidence suggests that acute exercise may be effective in significantly increasing blood catecholamine concentrations in healthy subjects, and that this increase may be dependent on exercise intensity [25, 26]. Moreover, it is more probable that it is related to increased secretion than to an impaired clearance or a decreased elimination [27]. Nevertheless, reliable studies aimed to assess the association between competitive endurance performance and markers of acute sympatho-adrenergic activation have been missing up until now. This is the first study that evaluates the effect of endurance exercise on plasma circulating metanephrines and their association with athlete’s running performance.

The measurement of metanephrines instead of their precursors, i.e., catecholamines, is usually considered more reliable for at least two reasons. First, plasma free metanephrines are conventionally regarded as more reliable biomarkers of adrenal medulla responsiveness to exercise compared to plasma catecholamines. The adrenal gland constitutes the single largest source of both MN and NMN, which contributes to approximately 90% and 23% of the circulating pool of these two hormones, respectively. On the other hand, plasma norepinephrine mainly derive from sympathetic nerves, whilst adrenal gland secretion only contributes for 7% of the circulating pool [28]. Plasma circulating metanephrines therefore seem to be a more suitable biomarker in assessing the contribution of adrenal medulla hypertrophy in predicting athletic performance. Then, the stability of plasma free metanephrines is much higher than that of catecholamines, and the former class of metabolites also has a more favorable pharmacokinetics profile [11]. Finally, metanephrines measurement has now completely replaced the assessment of plasma and urinary catecholamines for diagnosing and monitoring pheochromocytoma, as recommended by current guidelines [29], so that innovative methods have been specifically developed for allowing metanephrines quantification with high sensitivity and specificity [30].

Regarding the results of our study, the plasma concentrations of both MN and NMN were found to be significantly enhanced after a half-marathon run, despite the kinetics of both hormones being consistently different. The magnitude of increase of plasma NMN was more marked (i.e., almost double) than that of MN. Moreover, 6 hours after the end of the run the plasma concentration of MN was similar to that measured before the run, whilst plasma NMN levels persisted significantly increased even 24 hours after the end of the run.

Interestingly, both pre-run and delta value of NMN were significantly associated with running time. More specifically, better running performances were achieved by subjects with lower basal values and higher delta variation during the run, the latter case thus reflecting a higher secretion in response to exercise. These results, along with the significant correlation observed between delta variation of NMN and VO_2max_, suggests the existence of an association between endurance performance and improved adaptation of sympathoadrenal system. According to the “sport adrenal medulla” hypothesis, the training status of the athletes may be the reason of the observed data, since trained subjects might have decrease metanephrines release at rest and higher catecholamine secretion capacity compared to less trained individuals, thus providing them an adaptive advantage in terms of athletic performance [18]. Nevertheless, it is quite surprising that similar findings could not be replicated for MN. This hormone is almost entirely produced by the adrenal gland, whereas only 23% of the NMN circulating pool originates from the adrenal gland [28]. Therefore, factors other than training-induced adaptation of adrenal gland secretion capacity may come into play to explain the association between plasma NMN and endurance performance.

Important differences exist in the extra-neuronal production of MN and NMN [28]. The largest part of circulating MN (i.e., up to 90%) originates from the metabolism of locally released epinephrine, whilst a minor fraction (i.e., around 6%) is derived from the metabolism of circulating epinephrine. The spillover rate of MN originating from locally-released epinephrine is almost entirely attributable to adrenal gland production, whilst the amount of hormone derived from the metabolism of epinephrine removed from plasma is almost equally sustained by liver and the lung tissues. Unlike MN, the spillover rate of NMN originated from metabolism of locally-released norepinephrine approximates 80%. The individual adrenal medulla contributes to the largest part of this pool (i.e., 23%), whereas skeletal muscle, lung, kidney, mesenteric organs and liver almost contribute to the remaining fraction. Circulating norepinephrine metabolism contributes to 19% of total NMN production. Interestingly, the liver not only is the largest site of norepinephrine catabolism (57%), but also the organ which contribute most to the synthesis of NMN from metabolism of circulating norepinephrine (54%). Accordingly, adrenal gland and liver contribute to 23% and 20% of the overall circulating pool of NMN, respectively [28].

Due to heterogeneous pathways of metanephrines metabolism, and to the variable contribution to the overall MN and NMN spillover of various tissues and organs, the different association observed between endurance performance and each of these two hormones is not really surprising. We can therefore speculate that the biological interplay between NMN and running performance should be sought as an adaptive phenomenon occurring not only, and not primarily, in the adrenal gland, but more likely in other tissues, especially in the liver.

Symphatoadrenal system and catecholamines fluctuations are without doubt involved in cardiovascular, respiratory, metabolic and skeletal muscle adaptations induced by exercise. Although many aspects should be elucidated, this is the first study demonstrating a significant association between endurance performance and NMN, an extensively described marker of acute symphatoadrenal activation. Even more interestingly, a multivariate model incorporating variation of plasma NMN before and after the run, age, gender and capillary blood lactate could explain over 80% variance in running performance.

Notably, these results cannot be straightforwardly transferred to professional athletes, who are supposed to have higher values of stress-related hormones due to their intensive training, jet-lag, competitions stressors, and additional issues which can magnify the release of these molecules. We also acknowledge that more comprehensive information may have been generated by analyzing other stress hormones such as cortisol and ACTH. Although plasma MN and NMN are non-functional hormone, they both correlate with the respective catecholamines epinephrine and norepinephrine [31]. The measurement of metanephrines has several advantages over that of catecholamines, in that the former class of hormones is not significantly influenced by circadian rhythm, menstrual cycle or venipuncture. Metanephrines are also characterized by a longer half-life [32], are stable when stored at 4° C for 72 h after separation [33] and can now be measured with high sensitivity and specificity by LC-MS/MS [34]. Therefore, our preliminary findings pave the way for further studies aimed to define whether or not NMN assessment may provide valuable information for predicting endurance performance in the individual athlete and, eventually, validate this measure as a surrogate marker for early identification of overreaching and overtraining. It cannot also be excluded that subjects in overtraining might present a blunted metanephrines release in response to exercise similar to that already observed for other stress hormones such as prolactin, ACTH and growth hormone (GH), as a physiological response in the initial stages of relative glands dysfunction [35]. Therefore, additional studies should be planned to define whether or not metanephrines assessment may complement these measure for enabling an improved and earlier identification of both overreaching and overtraining.

The measurement of plasma metanephrines instead of their parent cathecolamines in further studies might be the key to resolve the great amount of inconsistent results reported in literature on the association between sympathoadrenal activation, competition performance and training status. Moreover, a definitive answer on the biological mechanism linking the association between NMN values and running performance will come from large prospective studies taking into account an appropriate monitoring of the individual training load in the preparation of competition.

## MATERIALS AND METHODS

The study was conducted during an event formally known as ‘Run For Science’, which took place in the town of Verona (Italy) in April 2014, and which was specifically planned to assess the individual response of recreational athletes to endurance running. Detailed information about this event has been previously published [36]. Briefly, in this study we enrolled 26 amateur runners who successfully concluded a 21.1 km (half-marathon) run between 75%–85% of their maximal oxygen uptake (VO_2max_). All these athletes were part of an amateur team and were engaged in habitual recreational running (median training regimen, 200 min/week). The VO_2max_ was measured before the event by means of a running test on a treadmill using a breath by breath ergospirometric system (Quark B2, Cosmed Italy). The trial started at 9.30 a.m. The run distance was covered on a relatively flat route, around the city of Verona (35 m vertical gain, maximal slope of 1.8%). The day was partially sunny, with temperatures comprised between 12–19° C and humidity comprised between 55–75%. All participants were allowed to drink ad libitum throughout the running distance, but were not allow to ingest carbohydrates.

Blood samples, obtained by venipuncture, were collected before the run and immediately afterwards in primary blood tubes containing either K_2_EDTA (for obtaining plasma EDTA) or no additives (for obtaining serum). Two additional plasma and serum samples were collected 6 and 24 hours after the end of the run. The samples were immediately transported to the laboratory of clinical biochemistry of the University Hospital of Verona, under controlled conditions of temperature (i.e., between 4–8° C). Immediately upon arrival in the laboratory, all blood tubes were centrifuged for 10 min at 1300 × g. The serum samples were then used for performing routine clinical chemistry testing, whilst plasma EDTA samples were aliquoted and stored at –80° C for later batch analysis of metanephrines. Capillary blood lactate was also assessed at the end of the run, from the ear lobe, using a Biosen C-Line Sport Analyzer (EKF Diagnostics, Magdeburg, Germany). The panel of serum analytes tested is reported in Supplementary Table 1, along with the pre- and post-run data. The analytical performance of all tests has been previously described elsewhere [37]. None of the participants in this study had taken medications the 2 days before the trial.

The quantification of plasma levels of MN and NMN was performed with a ClinMass Complete kit (Recipe, Munchen, Germany), by using liquid chromatoghraphy tandem mass spectrometry (LC-MS/MS). The LC-MS/MS analytical system was a Nexera X2 series UHPLC (Shimadzu, Kyoto, Japan) coupled with a 4500 MD triple quadrupole MS detector (Sciex, Milan, Italy). Sample processing was performed via solid phase extraction (SPE) which provided sample purification and enrichment in metanephrines content. The electrospray ionization was carried out in positive mode. Data were recorded in multiple reaction monitoring mode (MRM). Analyst 1.6.2. and Multiquant 3.0.2. softwares (Sciex) were used for data acquisition and quantification, respectively. The method was found to be linear up to 100000 pmol/L for both metanephrines and was characterized by a limit of quantitation (LOQ) of 20 pmol/L. The mean intra- and inter-assay imprecision, as assessed using three plasma pools with different metanephrines concentrations, were comprised between 1.7–3.9% for MN and between 3.2–6.2% for NMN, respectively.

Demographical, physiological and ergonomic data of the participants are shown in Table 1. Normal distribution of variables was tested by Kolmogorov-Smirnov test. Differences between pre- and post-run values were analyzed with Wilcoxon’s test for paired samples or paired Student’s *T*-test, when appropriate. Adjustment for body weight change throughout the study period did not generate significantly different results, and was hence disregarded (data not shown). Pearson’s correlation was used to test the association between metanephrines delta value (i.e., the percentage difference between pre- and post-run values), ergonomic and laboratory values. Univariate and multivariate linear regression analyses were also conducted for identifying variables significantly associated with running performance. Results are shown as median and range.

The statistical analysis was performed with Analyse-it (Analyse-it Software Ltd, Leeds, UK). Figures were achieved by using GraphPad Prism 5.01 (San Diego, California). All subjects provided a written consent for being enrolled. The study was approved by the local Ethical Committee (Department of Neurological, Neuropsychological, Morphological and Movement Sciences, University of Verona).

## SUPPLEMENTARY MATERIALS TABLE



## References

[1] Zouhal H, Jacob C, Delamarche P, Gratas-Delamarche A (2008). Catecholamines and the effects of exercise, training and gender. Sports Med.

[2] Kjaer M, Mikines KJ, Christensen NJ, Tronier B, Vinten J, Sonne B, Richter EA, Galbo H (1984). Glucose turnover and hormonal changes during insulin-induced hypoglycemia in trained humans. J Appl Physiol Respir Environ Exerc Physiol.

[3] Silva TM, Takakura AC, Moreira TS (2016). Acute hypoxia activates hypothalamic paraventricular nucleus-projecting catecholaminergic neurons in the C1 region. Exp Neurol.

[4] Svatikova A, Covassin N, Somers KR, Somers KV, Soucek F, Kara T, Bukartyk J (2015). A Randomized Trial of Cardiovascular Responses to Energy Drink Consumption in Healthy Adults. JAMA.

[5] Sanchis-Gomar F, Leischik R, Lippi G (2016). Energy drinks: Increasing evidence of negative cardiovascular effects. Int J Cardiol.

[6] Von Euler US, Hellner S (1952). Excretion of noradrenaline and adrenaline in muscular work. Acta Physiol Scand.

[7] Cryer PE (1980). Physiology and pathophysiology of the human sympathoadrenal neuroendocrine system. N Engl J Med.

[8] McCorry LK (2007). Physiology of the autonomic nervous system. Am J Pharm Educ.

[9] Perez-Quilis C, Kingsley JD, Malkani K, Cervellin G, Lippi G, Sanchis-Gomar F (2017). Modulation of heart rate by acute or chronic aerobic exercise. Potential effects on blood pressure control. Curr Pharm Des.

[10] Leosco D, Parisi V, Femminella GD, Formisano R, Petraglia L, Allocca E, Bonaduce D (2013). Effects of exercise training on cardiovascular adrenergic system. Front Physiol.

[11] Raber W, Raffesberg W, Bischof M, Scheuba C, Niederle B, Gasic S, Waldhäusl W, Roden M (2000). Diagnostic efficacy of unconjugated plasma metanephrines for the detection of pheochromocytoma. Arch Intern Med.

[12] Eisenhofer G, Lenders JW, Linehan WM, Walther MM, Goldstein DS, Keiser HR (1999). Plasma normetanephrine and metanephrine for detecting pheochromocytoma in von Hippel-Lindau disease and multiple endocrine neoplasia type 2. N Engl J Med.

[13] Dorn GW, Force T (2005). Protein kinase cascades in the regulation of cardiac hypertrophy. J Clin Invest.

[14] Kovacs R, Baggish AL (2016). Cardiovascular adaptation in athletes. Trends Cardiovasc Med.

[15] Sharma S, Merghani A, Mont L (2015). Exercise and the heart: the good, the bad, and the ugly. Eur Heart J.

[16] Wasfy MM, Weiner RB (2015). Differentiating the athlete’s heart from hypertrophic cardiomyopathy. Curr Opin Cardiol.

[17] Forteza-Alberti JF, Sanchis-Gomar F, Lippi G, Cervellin G, Lucia A, Calderon-Montero FJ (2017). Limits of ventricular function: from athlete’s heart to a failing heart. Clin Physiol Funct Imaging.

[18] Kjaer M (1998). Adrenal medulla and exercise training. Eur J Appl Physiol Occup Physiol.

[19] Ostman I, Sjostrand NO (1971). Effect of prolonged physical training on the catecholamine levels of the heart and the adrenals of the rat. Acta Physiol Scand.

[20] Schmidt KN, Gosselin LE, Stanley WC (1992). Endurance exercise training causes adrenal medullary hypertrophy in young and old Fischer 344 rats. Horm Metab Res.

[21] Knopfli B, Calvert R, Bar-Or O, Villiger B, Von Duvillard SP (2001). Competition performance and basal nocturnal catecholamine excretion in cross-country skiers. Med Sci Sports Exerc.

[22] Pierce D, Kupprat I, Harry D (1976). Urinary epinephrine and norepinephrine levels in women athletes during training and competition. Eur J Appl Physiol Occup Physiol.

[23] Lehmann M, Jakob E, Roscher E, Tusch R, Keul J (1988). Ski-flying: related catecholamine excretion compared with cross-country skiing. Int J Sports Med.

[24] Svensson E, Thanderz MA, Sjoberg L, Gillberg M (1988). Military flight experience and sympatho-adrenal activity. Aviat Space Environ Med.

[25] Galbo H, Holst JJ, Christensen NJ (1975). Glucagon and plasma catecholamine responses to graded and prolonged exercise in man. J Appl Physiol.

[26] Kjaer M, Secher NH, Bach FW, Galbo H (1987). Role of motor center activity for hormonal changes and substrate mobilization in humans. Am J Physiol.

[27] Kjaer M, Christensen NJ, Sonne B, Richter EA, Galbo H (1985). Effect of exercise on epinephrine turnover in trained and untrained male subjects. J Appl Physiol (1985).

[28] Eisenhofer G, Rundquist B, Aneman A, Friberg P, Dakak N, Kopin IJ, Jacobs MC, Lenders JW (1995). Regional release and removal of catecholamines and extraneuronal metabolism to metanephrines. J Clin Endocrinol Metab.

[29] Lenders JW, Duh QY, Eisenhofer G, Gimenez-Roqueplo AP, Grebe SK, Murad MH, Naruse M, Pacak K, Young WF, Endocrine Society (2014). Pheochromocytoma and paraganglioma: an endocrine society clinical practice guideline. J Clin Endocrinol Metab.

[30] Heideloff C, Payto D, Wang S (2016). Quantitation of Free Metanephrines in Plasma by Liquid Chromatography-Tandem Mass Spectrometry. Methods Mol Biol.

[31] Roden M, Raffesberg W, Raber W, Bernroider E, Niederle B, Waldhausl W, Gasic S (2001). Quantification of unconjugated metanephrines in human plasma without interference by acetaminophen. Clin Chem.

[32] Campbell KA, Joseph SP, Whiting MJ, Doogue MP (2012). The half-lives of plasma free metanephrines. Clin Endocrinol (Oxf).

[33] Deutschbein T, Unger N, Jaeger A, Broecker-Preuss M, Mann K, Petersenn S (2010). Influence of various confounding variables and storage conditions on metanephrine and normetanephrine levels in plasma. Clin Endocrinol (Oxf).

[34] de Jong WH, Graham KS, van der Molen JC, Links TP, Morris MR, Ross HA, de Vries EG, Kema IP (2007). Plasma free metanephrine measurement using automated online solid-phase extraction HPLC tandem mass spectrometry. Clin Chem.

[35] Cadegiani FA, Kater CE (2017). Hormonal aspects of overtraining syndrome: a systematic review. BMC Sports Sci Med Rehabil.

[36] Lippi G, Schena F (2017). Run for Science (R4S): the history of a successful project of precision and laboratory medicine in sport and exercise. J Lab Precis Med.

[37] Lippi G, Salvagno GL, Danese E, Tarperi C, La Torre A, Guidi GC, Schena F (2015). The baseline serum value of alpha-amylase is a significant predictor of distance running performance. Clin Chem Lab Med.

