# Supplementary material for: Sympatho-adrenergic activation by endurance exercise: Effect on metanephrines spillover and its role in predicting athlete’s performance
Source: Oncotarget. 2018 Feb 26;9(21):15650–7. doi: 10.18632/oncotarget.24584 (PMC5884654; doi:10.18632/oncotarget.24584)
Supplement: Supplementary file 1 [file oncotarget-09-15650-s001.pdf]

Sympatho-adrenergic activation by endurance exercise: Effect on metanephrines spillover and its role in predicting athlete's performance

SUPPLEMENTARY MATERIALS

Supplementary Table 1: Pre- and post-run clinical chemistry analyses

	Pre-run	Post-run	*p*-value
Capillary blood lactate (mmol/L)	–	3.75 (2.22–9.39)	
Albumin (g/L)	46 (41–53)	48.1 (43–54.4)	**<0.001**
Total protein (g/L)	76.7 (67.3–84.5)	77.2 (68.3–88.8)	**0.004**
Creatinine (umol/L)	84 (60–105)	120 (81–139)	**<0.001**
eGFR (mL/min/1.73m^2)	76 (63–93)	54 (41–67)	**<0.001**
Urea (mmol/L)	5.79 (3.43–7.57)	6.53 (3.79–9.61)	**<0.001**
Bilirubin (µmol/L)	8.35 (3.2–17.6)	12.9 (5.5–23.9)	**<0.001**
Calcium (mmol/L)	2.30 (2.09–2.52)	2.33 (2.17–2.58)	**0.004**
Chloride. mmol/L	104 (100–109)	104 (101–108)	0.716
Potassium (mmol/L)	4.42 (3.93–4.73)	4.2 (3.88–4.89)	0.197
Sodium (mmol/L)	141 (137–144)	144 (138–146)	**<0.001**
Total cholesterol (mmol/L)	5.23 (4.22–7.05)	5.08 (4.08–6.92)	0.798
HDL-cholesterol (mmol/L)	1.85 (1.22–3.5)	1.96 (1.38–3.31)	**0.016**
Triglycerides (mmol/L)	0.85 (0.50–1.94)	1.03 (0.67–1.92)	**0.038**
Iron (µmol/L)	17.5 (4.0–29)	21 (6–39)	**<0.001**
Glucose (mmol/L)	4.5 (3.7–6.2)	4.4 (3.4–7.5)	0.470
AST (U/L)	29 (19–43)	34 (24–46)	**<0.001**
ALT (U/L)	21 (15–33)	23 (16–37)	**<0.001**
GGT (U/L)	19 (9–65)	18 (9–59)	0.422
ALP (U/L)	56.5 (42–79)	60 (44–90)	**0.001**
Uric acid (µmol/L)	282 (156–450)	340 (189–557)	**<0.001**
α-amylase (U/L)	84 (35–216)	84 (38–170)	0.497
Lipase (U/L)	32 (14–439)	32 (12–167)	0.378
LDH (U/L)	355 (261–574)	460 (368–589)	**<0.001**
CK (U/L)	161 (53–389)	224 (86–648)	**<0.001**

Abbreviations: ALP, alkaline phosphatase; ALT, alanine aminotransferase; AST, aspartate aminotransferase; BMI, body mass index; CK, creatine kinase; eGFR, estimated glomerular filtration rate; GGT, gamma-glutamyl transferase; HDL, high-density lipoprotein; LDL, low-density lipoprotein.
